# Effects of hypoxia on uteroplacental and fetoplacental vascular function during pregnancy

**DOI:** 10.3389/fphys.2024.1490154

**Published:** 2024-12-18

**Authors:** Germán A. Arenas, Ramón A. Lorca

**Affiliations:** Division of Reproductive Sciences, Department of Obstetrics and Gynecology, University of Colorado Anschutz Medical Campus, Aurora, CO, United States

**Keywords:** hypoxia, pregnancy, uterine artery, placenta, vascular reactivity

## Abstract

During pregnancy, marked changes in vasculature occur. The placenta is developed, and uteroplacental and fetoplacental circulations are established. These processes may be negatively affected by genetic anomalies, maternal environment (i.e., obesity or diabetes), and environmental conditions such as pollutants and hypoxia. Chronic hypoxia has detrimental effects on the vascular adaptations to pregnancy and fetal growth. The typical pregnancy-dependent rise in uterine blood flow by vascular remodeling and vasodilation of maternal uterine arteries is reduced, leading to increases in vascular tone. These maladaptations may lead to complications such as fetal growth restriction (FGR) and preeclampsia. In this review, the effect of hypoxia on uteroplacental and fetoplacental circulation and its impact on pregnancy outcomes in humans and animal models are discussed. Evidence is provided for several mechanisms that affect pregnancy through hypoxia-induced alterations. Future directions to fill gaps in knowledge and develop therapeutic strategies to prevent or alleviate hypoxia-related pregnancy complications, such as FGR and preeclampsia, are suggested.

## 1 Introduction

### 1.1 Uteroplacental and fetoplacental circulation during healthy pregnancy

Mammalian pregnancy causes profound and progressive adaptations in the maternal cardiovascular system intended to sustain the developing fetus. Maternal cardiac output is increased, arterial blood pressure is decreased, and peripheral vascular resistance is reduced, among other changes (Mahendru et al., 2014). Importantly, decreased vascular resistance leads to increased blood flow directed to the uterine circulation (Ford, 1982; Palmer et al., 1992; Konje et al., 2001), which is responsible for nutrient and gas exchange between the mother and the fetus through the placenta. Any interruption to these adaptations can result in suboptimal pregnancy outcomes or complications.

**FIGURE 1:**
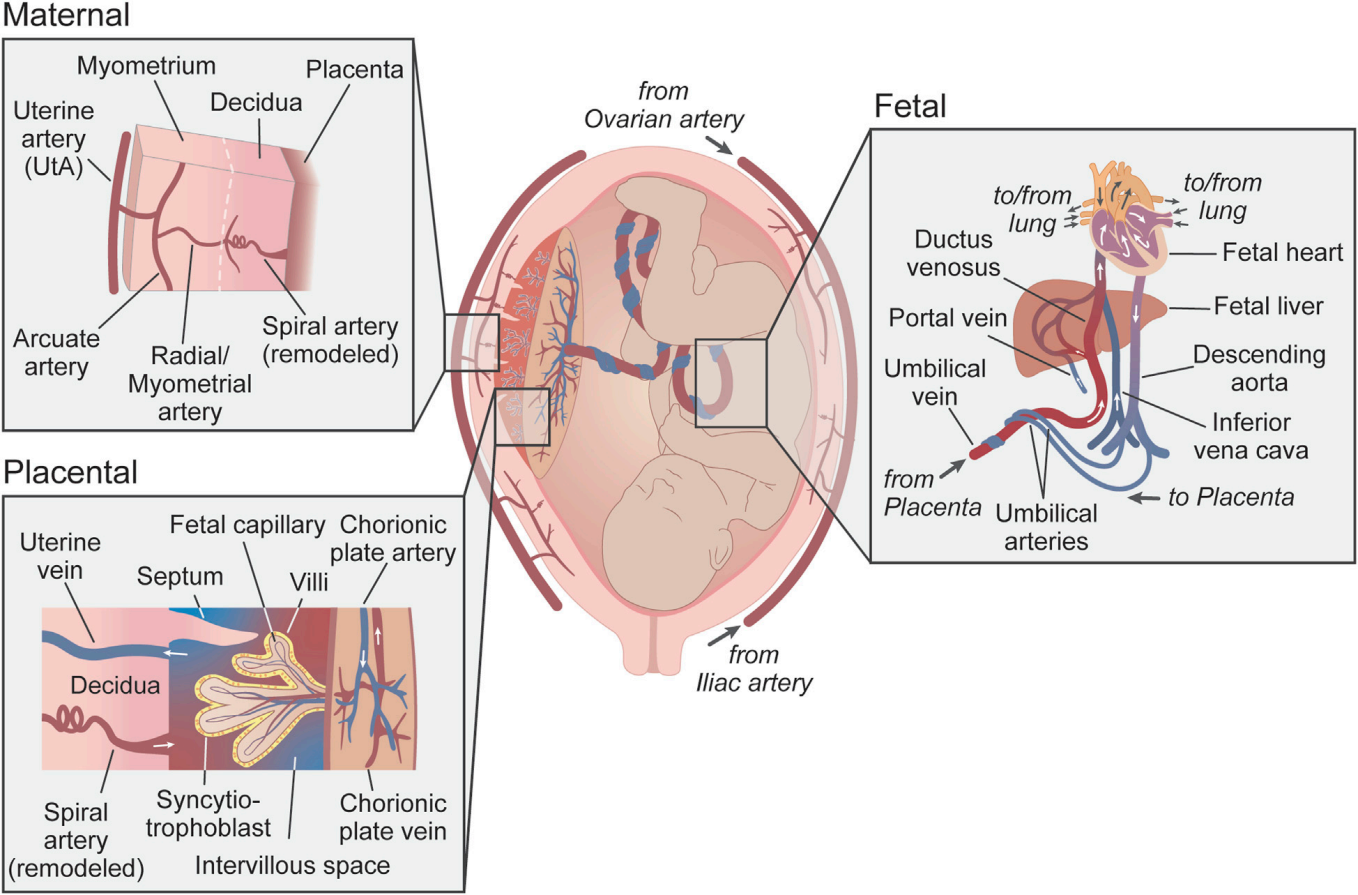
Uteroplacental and fetoplacental circulations. Schematic representation of the uteroplacental and fetoplacental blood vessels during pregnancy. Insets show a more detailed anatomical organization of the circulatory systems (maternal, placental, and fetal). Red vessels represent oxygenated blood, blue is non-oxygenated blood, and purple is mixed blood. Arrows show the direction of blood flow. Uterine veins and venules have been omitted from the maternal inset for simplification.

The circulation between the mother and the fetus can be divided into maternal, placental, and fetal compartments (Figure 1). In the human maternal vascular component, the main uterine arteries (UtAs) bifurcate from the bilateral internal iliac arteries. UtAs run along the serosal surface of the uterus and provide most of its blood supply, although there is also contribution from the ovarian arteries. Branching off the main UtAs are the arcuate arteries, which are parallel to, and embedded in the uterine smooth muscle layer, called the myometrium. Radial arteries (also known as myometrial arteries) branch off from the arcuate arteries towards the endometrium (or *decidua* in the pregnant state) – the inner layer of the uterus – where some end in a specialized coiled shape and are called spiral arteries (Degner et al., 2017; James et al., 2017). In non-pregnant individuals, the spiral arteries remain coiled and relatively constricted, as the demand for blood to the endometrium is small. However, during pregnancy, the spiral arteries near the embryo implantation site are invaded by placental trophoblast cells and remodeled to become wider and lower resistance to meet the blood flow demanded by the developing fetus (Pijnenborg et al., 2006; Zhang et al., 2023). Once uteroplacental circulation is established, the placental vascular component comes in contact with the maternal blood, which bathes the intervillous space of the placenta, where the syncytiotrophoblast cells allow the transport of gases and nutrients to and from the fetal circulation. In the fetal vascular component, the fetal internal iliac arteries connect to two umbilical arteries which run through the umbilical cord into the chorionic plate of the placenta. These arteries bifurcate into smaller branches (the chorionic plate arteries) and deeper into the chorionic villi to generate the fetal capillaries that will, in turn, become venules leaving the villous tree and converging into the chorionic plate veins and the umbilical vein (Chappell et al., 2023). The umbilical vein carries oxygenated blood and nutrients to the fetal circulation via the ductus venosus and small portal sinus (Basta and Lipsett, 2024; Remien and Majmundar, 2023) (Figure 1).

### 1.2 Maternal vascular changes during pregnancy

Early in human pregnancy, the embryo implants into the endometrium, which is then named decidua basalis, but uteroplacental circulation is not established until the end of the first trimester. Around this time, placental extravillous trophoblast cells migrate into the maternal spiral arteries, through somatic tissue and vessel lumen, and enlarge these blood vessels by replacing the endothelial cells (ECs), dedifferentiating smooth muscle cells (SMCs), and increasing vasodilation (Burton and Jauniaux, 2018; Ma et al., 2021).

The major changes to the maternal vasculature are both structural and functional. Structurally, the UtA undergoes remodeling, evidenced as an increase in diameter with species-dependent changes in wall thickness (Hilgers et al., 2003; Osol and Moore, 2014). There are several mechanisms for this remodeling, including cell hypertrophy, hyperplasia, and extracellular matrix remodeling (Osol and Moore, 2014). Assuming blood flow to be laminar, this increase in UtA diameter largely leads to increased blood flow as determined by Poiseuille’s law for laminar flow (Equation 1), in which the volumetric flow rate (*Q*) increases with the fourth power of the radius (*r*). Increased length (*l*) of UtA and uterine veins is inversely linearly related to *Q*, which is particularly important in multiparous animals. Both viscosity of the fluid (*η*) and pressure gradient (Δ*P*) are also linearly associated with *Q*.

$${Poiseuille}^{\prime }s\;law\;for\;laminar\;flow.\;Q=\frac{\pi \;\Delta P\;r^{4}}{8\eta l}$$ (1)

Functionally, UtAs exhibit decreased vasoconstriction and increased vasodilatory responses, which may contribute to the pregnancy-dependent rise in blood flow, as vessel diameter increases and resistance decreases. Several endothelial and vascular smooth muscle factors contribute to this regulation of vasoreactivity. Increased production of nitric oxide (NO), via endothelial NO synthase (eNOS), is elicited by estrogen, shear stress, vascular endothelial growth factor, or other mechanisms in ECs (Bird et al., 2003; Luksha et al., 2010). Whereas, in SMCs, increased K^+^ channel-dependent hyperpolarization is an important contributor to the reduced UtA vascular tone and increased diameter observed during pregnancy (Bresnitz and Lorca, 2022).

### 1.3 Impairments in vascular adaptations during pregnancy

Appropriately timed pregnancy-dependent changes in vasculature are critical for healthy pregnancy outcomes. Thus, impaired vascular adaptations are associated with several pregnancy complications, such as fetal growth restriction (FGR) and preeclampsia, which exhibit a blunted rise in UtA blood flow (Konje et al., 2003; Julian et al., 2008; Browne et al., 2011). For instance, human myometrial arteries from FGR pregnancies have less vasodilatory response than appropriate for gestational age (AGA) controls (Ong et al., 2003; Lorca et al., 2020), which is consistent with UtAs in animal models of FGR (Aljunaidy et al., 2016). Moreover, pregnant eNOS knockout mice develop FGR and are associated with impaired UtA function, showing higher vasoconstriction and impaired vasodilation compared to wild-type mice (Kusinski et al., 2012). The same mouse model also showed increased UtA resistance associated with structural and cellular changes contributing to dysregulated uteroplacental blood flow during pregnancy (van der Heijden et al., 2005; Rennie et al., 2015). Similarly, preeclampsia reduces the vasodilatory responses in the UtAs and myometrial arteries (Cockell and Poston, 1997; Kublickiene et al., 2000; Luksha et al., 2010). Although the etiology of preeclampsia is likely multifactorial, a common contributor to this pregnancy complication is a shallow invasion of the maternal spiral arteries by the extravillous trophoblast. This prevents the proper remodeling of the spiral arteries and impairs the normal function of the placenta (Burton et al., 2009). Maternal endothelial progenitor cells may also contribute to the etiology of preeclampsia insofar as endothelial progenitor cells from preeclamptic pregnancies promote a transition of spiral artery SMCs into a synthetic phenotype that accumulates extracellular matrix components before trophoblast invasion/remodeling, ultimately contributing to reduced uteroplacental perfusion (Tan et al., 2024). Other insults that reduce UtA blood flow, such as surgical ligations of the uterine vessels or exposure to hypoxic conditions, are often utilized in animal models of these pregnancy complications (Alexander et al., 2001; Vuguin, 2007; Janot et al., 2014; Aljunaidy et al., 2016; Lane et al., 2020c).

### 1.4 Effect of hypoxia on systemic vascular function

Hypoxia is a strong driver of vascular reactivity, inducing constriction in pulmonary arteries and vasodilation in systemic arteries. These diverse physiological effects of hypoxia respond to the need to preserve gas exchange in the lungs and promote blood delivery to systemic tissues under low oxygen conditions. Systemic arteries dilate in response to hypoxia via multiple mechanisms. Specifically, hypoxia produces a decrease in ATP levels, activation of ATP-sensitive K^+^ (K_ATP_) channels, and reduction in intracellular Ca^2+^ levels in smooth muscle, leading to dilation (Taggart and Wray, 1998). Chronic hypoxia also elicits vascular remodeling. For instance, in pulmonary arteries, high-altitude residence and animals exposed to artificial ambient hypoxic conditions thicken the arterial wall and increase the production of extracellular matrix proteins (Arias-Stella and Saldana, 1963; Rabinovitch et al., 1979; Heath et al., 1981; Wang and Chesler, 2012).

## 2 Maternal vascular dysfunction induced by hypoxia

**FIGURE 2:**
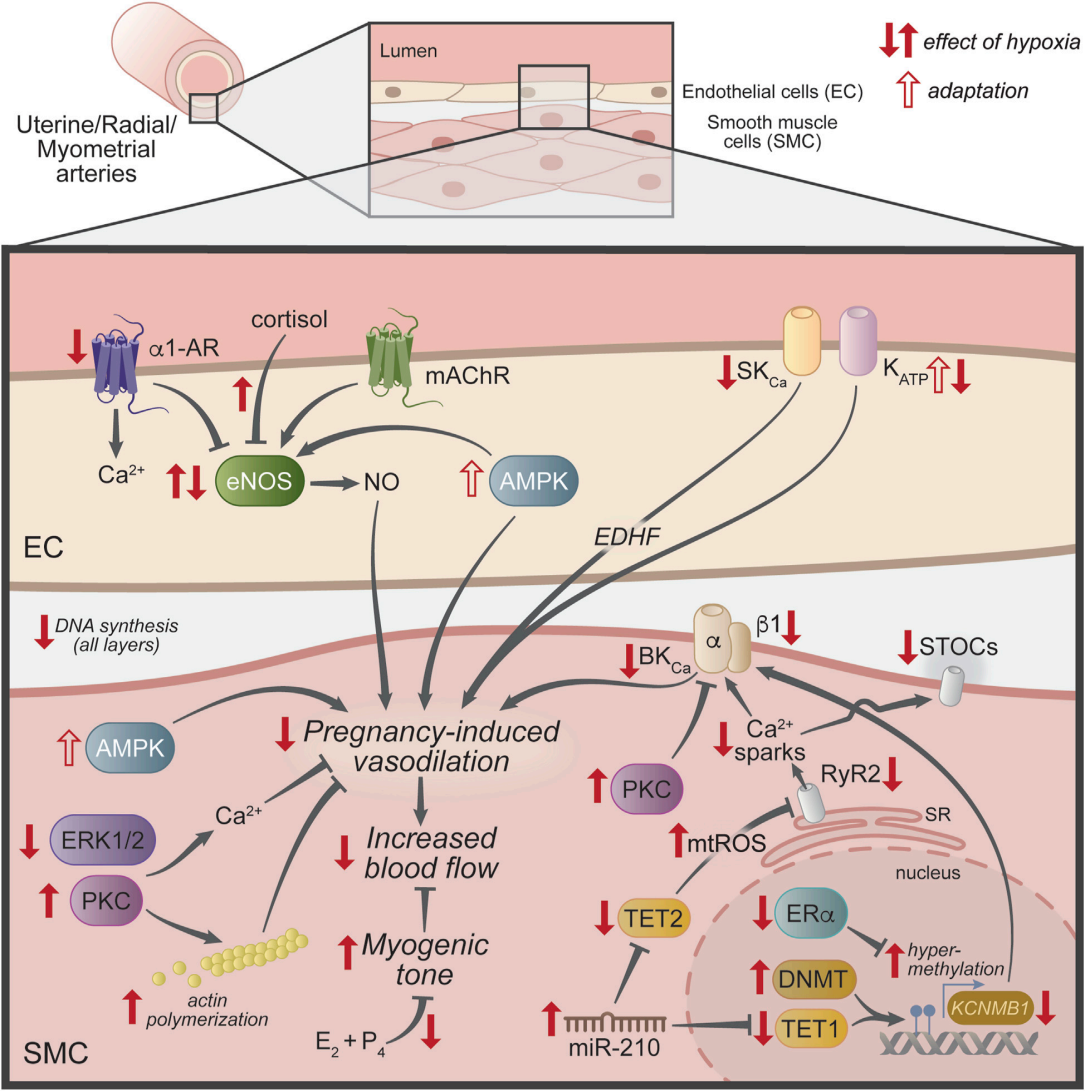
Mechanisms underlying the effect of hypoxia in uterine vasculature. Schematic representation of uterine vascular regulation mechanisms that are modified by hypoxia (filled red arrows) during pregnancy to reduce the rise in uterine blood flow. The open red arrows show mechanisms thought to be compensatory or adaptions to the lack of oxygen. Abbreviations: α1-AR, alpha-1 adrenergic receptor; AMPK, AMP-activated protein kinase; BK_Ca_, large-conductance Ca^2+^- activated K^+^ channel; DNMT, DNA methyltransferase; E_2_, 17β-estradiol; EC, endothelial cell; EDHF, endothelial-derived hyperpolarizing factor; eNOS, endothelial nitric oxide synthase; ERα, estrogen receptor-α; ERK1/2, extracellular signal-regulated kinase 1/2; K_ATP_, ATP-sensitive K^+^ channel; *KCNMB1*, large-conductance Ca^2+^-activated K^+^ channel β1 subunit gene; mAChR, muscarinic acetylcholine receptor; mir-210, micro RNA 210; mtROS, mitochondrial reactive oxygen species; NO, nitric oxide; P_4_, progesterone; PKC, protein kinase C; RyR1/2, ryanodine receptors 1 and 2; SK_Ca_, small-conductance Ca^2+^-activated K^+^ channel; SMC, smooth muscle cell; SR, sarcoplasmic reticulum; STOCs, spontaneous transient outward currents; TET1 and 2, ten-eleven translocation methylcytosine dioxygenase 1 and 2.

In uterine vasculature, hypoxia decreases vasodilation and increases vascular tone by multiple mechanisms (Figure 2). Hypoxia is a necessary trait during early mammalian development to prevent excess reactive oxygen species (ROS) during the highly proliferative early pregnancy stages. However, prolonged exposure to low oxygen levels during pregnancy can lead to FGR (Jensen and Moore, 1997; Julian et al., 2008) and preeclampsia (Palmer et al., 1999; Moore, 2021). Hypoxia-induced uterine vascular dysfunction is characterized by alterations in hemodynamic parameters. Several reports have shown increased UtA resistance and decreased UtA diameter reduce blood flow in women living at high altitudes (defined as elevations above 2500 m) compared to low altitudes (Zamudio et al., 1995; Krampl et al., 2001; Julian et al., 2008). Reduced UtA blood flow in high-altitude pregnancies is attributed, in part, to a high endothelin-1/NO metabolites ratio (Julian et al., 2008). Similarly, chronic hypoxia-exposed pregnant guinea pigs show increased UtA pulsatility and resistance indexes (Turan et al., 2017) and decreased UtA capacity for growth during pregnancy. These responses to hypoxia are due to modifications in the proliferative response of vascular SMCs *in vitro* (Rockwell et al., 2006) and compromised biomechanical properties of the UtA, such as increased blood vessel distensibility and an altered stress-strain relationship (Mateev et al., 2006). The similar effects of hypoxia on maternal UtA blood flow in humans and guinea pigs could be a result of the similarities in their pregnancies, as both species develop haemomonochorial placentas, deliver precocial neonates, present similar uterine blood flow distribution throughout pregnancy, and fetal growth and development (Morrison et al., 2018; Carter, 2020; Candia et al., 2023).

Rats exposed to chronic hypoxia during pregnancy also exhibit reduced endothelium-dependent vasodilatory responses, however, their UtA pulsatility and resistance indexes are decreased (Aljunaidy et al., 2016). Likewise, mice exposed to chronic hypoxia during pregnancy show an increase or no change in UtA blood flow compared to normoxic mice (Lane et al., 2020b; Lane et al., 2020c). This hypoxia-induced increase in UtA blood flow in mice and rats, opposite humans and guinea pigs, may be a compensatory mechanism, although it is insufficient to prevent hypoxia-induced FGR (Aljunaidy et al., 2016; Lane et al., 2020b). This compensatory mechanism could be associated with the higher tolerance to hypoxia observed in rodents compared to humans. Mice and rats typically have the ability to develop higher tissue capillary density, can decrease their body temperature, and increase their whole-body oxygen consumption as adaptive mechanisms to resist hypoxia (Rising and D'Alecy, 1989; Dzhalilova and Makarova, 2020; Arias-Reyes et al., 2021). Hence, rodent models must be exposed to more severe hypoxic conditions than humans to create comparable stressors. Another explanation for these differences between mice and rats with humans and guinea pigs could be due to the distinct placental structures among these species: mice and rats develop a haemotrichorial placenta (two syncytiotrophoblast layers and one cytotrophoblast layer) as opposed to the haemomonochorial structure observed in humans and guinea pigs (Georgiades et al., 2002).

Specific cellular and molecular mechanisms known to underlie the hypoxia-induced reduction of uteroplacental perfusion are discussed below.

### 2.1 Estrogen and other steroid hormones

Estrogen, acting on estrogen receptor-α (ERα) and estrogen receptor-β (ERβ), is a regulator of vascular function in different vascular beds (Rubanyi et al., 2002). ERα and ERβ modulate the transcription of several genes due to the direct interaction of the ER complex with estrogen response elements (Albrecht and Pepe, 1990; Mahendru et al., 2014). In human UtAs, increased expression of ERα correlates inversely with collagen levels and distensibility of these arteries (Lydrup and Ferno, 2003). During pregnancy, the dramatic increase in circulating estrogen levels contributes to the increase in UtA blood flow (Li et al., 2022). Increased ERα expression leads to greater binding of circulating estrogen, which induces the transcription of eNOS, with a consequent increase in NO production in the UtA endothelium (Magness et al., 2001). Estrogen also binds to membrane receptors, which activate UtA vasodilator pathways mediated mainly by acute activation of eNOS (Chen et al., 2004).

Chronic hypoxia prevents the normal pregnancy- and sex steroid-induced increase in ERα expression in the pregnant ovine UtA (Xiao et al., 2009; Chang et al., 2010). Mechanistically, hypoxia induces epigenetic modifications (i.e., DNA methylation and histone modification) that repress ERα gene expression in the UtA (Dasgupta et al., 2012; Chen et al., 2015). In DNA methylation, a methyl group binds to cytosine residues in CpG sequences, which can suppress gene transcription by rendering the DNA unrecognizable in response to the binding of some transcription factors (Handy et al., 2011; Fuentes and Silveyra, 2019). Hypoxia has been reported to induce CpG methylation at a wide range, including repression of ERα (Chen et al., 2015). In ovine UtA, ERα gene repression is mediated through increased promoter methylation at critical transcription factor binding sites, such as specific protein 1 and upstream stimulatory factor, reducing ERα promoter activity (Dasgupta et al., 2012). Thus, the hypoxia-induced repression of ERα gene expression impairs normal vascular adaptations to pregnancy, largely by epigenetic modulation.

ERβ also contributes to UtA vasodilation during pregnancy. ERβ is upregulated during pregnancy in endothelial and vascular smooth muscle cells in the UtA of pregnant ewes (Byers et al., 2005; Liao et al., 2005). In UtA endothelial cells, specific activation of ERβ alone induces a decrease in the inhibitory site of eNOS (Thr495), leading to elevated levels of NO, similarly to the effect of ERα expression (Pastore et al., 2016). In addition, increased ERβ during pregnancy mediates upregulation of the angiotensin II type 2 receptor (AT2R) expression in UtA endothelium via transactivation of the AT2R promoter (Mishra et al., 2019), and AT2R activation increases UtA blood flow in rats (Mishra et al., 2018). In addition, pharmacological activation of ERβ by diarylpropionitrile reduces protein kinase C (PKC)-dependent maximal vasoconstriction in UtA isolated from pregnant ewes (Chang et al., 2010). In the same study, the authors showed that ERβ abundance in the UtA does not change in hypoxic pregnant ewes compared to low-altitude controls (Chang et al., 2010), suggesting that ERβ is not modulated by gestational chronic hypoxia.

Progesterone (P_4_) treatment of sheep UtA upregulates eNOS expression in non-pregnant ewes, and this effect is enhanced in the presence of 17β-estradiol (E_2_) (Rupnow et al., 2001). Treatment with both steroid hormones (P_4_+E_2_), at levels similar to those observed during pregnancy, decreases myogenic tone in non-pregnant UtA. In contrast, selective blockade of their receptors induces an increase in UtA myogenic tone (Xiao et al., 2009). Notably, this sex steroid hormone-dependent reduction of UtA myogenic tone is blunted by chronic exposure to hypoxia during gestation (Chang et al., 2010).

Cortisol may also play a role in the regulation of UtA vascular tone. Although maternal cortisol has been shown to be elevated during pregnancy (Nolten and Rueckert, 1981), its effects on the uterine vasculature remain unclear. Studies performed in UtA from non-pregnant ewes treated with cortisol showed a potentiation in the constrictor response to norepinephrine (NE) and a decrease in eNOS expression, contributing to a pro-constrictor state (Xiao et al., 2002). However, reducing endogenous cortisol levels via adrenalectomy in non-pregnant ewes also reduces eNOS expression in the UtA (Li et al., 2007). Pregnancy decreases the cortisol-induced increase in UtA vasoconstriction and reduces by half the cortisol-dependent downregulation in eNOS expression (Xiao et al., 2002). Notably, pregnant ewes exposed to acute cortisol treatment show no difference in UtA flow compared to untreated controls (Vaughan et al., 2016). A prospective cohort study demonstrated an association between high maternal cortisol levels and low birth weight (Shriyan et al., 2023), suggesting a relationship between cortisol-dependent UtA constriction and birth weight. Further studies are required to determine the precise role of cortisol in regulating UtA vasoreactivity during pregnancy. In relation to hypoxia, one study has shown that chronic hypoxic exposure during pregnancy increases cortisol sensitivity in the UtA, which contributes to a pro-constrictor state (Xiao et al., 2004).

### 2.2 Nitric oxide (NO) signaling

Healthy pregnancy typically involves an increase in NO-dependent vasodilation of UtA (White et al., 2000). In one study, pregnant women exposed to chronic hypoxia due to high-altitude residency show decreased vasodilatory responses to acetylcholine in myometrial arteries compared to low-altitude residents. The reduction was caused by decreased NO signaling, but eNOS expression was unchanged (Lorca et al., 2019), suggesting that the reduced effect of NO could be upstream of eNOS (i.e., at the cholinergic receptor or its coupling to Ca^2+^ signaling) or downstream of eNOS (i.e., cyclic GMP signaling, nitrosylation of target proteins, etc.). In pregnant animal models, chronic hypoxia impairs UtA flow-dependent vasodilator responses (Mateev et al., 2003) and decreases NO-dependent vasodilation (White et al., 2000). Intermittent hypoxia in pregnant mice also impairs NO-mediated UtA vasodilation (Badran et al., 2019). In contrast, chronic hypoxia has been found to lead to enhanced NO-mediated vasodilation and increased eNOS expression in UtA from pregnant ewes (Xiao et al., 2001), which contributes to increased endothelium-dependent vasodilator signaling. Interestingly, pregnant ewes exposed to high-altitude chronic hypoxia from these studies do not develop FGR (Kamitomo et al., 1992), unlike pregnant sheep studied at other high-altitude locations (Herrera et al., 2007) or other animals such as mice, rats, or guinea pigs (Aljunaidy et al., 2016; Turan et al., 2017; Lane et al., 2020b). This difference among species may be due to the hypoxia-evoked increase in NO response observed in UtAs from the pregnant ewes used in these studies (Xiao et al., 2001). In sheep studies that observed FGR development (Herrera et al., 2007), differences in fetal growth induced by hypoxia could be due to specific responses by different breeds of sheep or environmental and/or nutritional differences.

### 2.3 Adrenergic signaling

Vasoconstrictor responses to adrenergic stimulation in pregnancy are species-specific. Studies conducted in pregnant ovine UtA showed increased sensitivity to α1-adrenergic receptor stimulation with NE compared to non-pregnant sheep (Xiao et al., 2002). In humans, pregnant UtA also showed increased sensitivity to NE when compared to UtA from non-pregnant women (Rosenfeld et al., 2012). UtA from late-pregnant rats showed three-fold increases in vasoconstrictor responses to the α-1 agonist phenylephrine (PE) compared to non-pregnant rats (Osol and Cipolla, 1993). However, in pregnant guinea pig UtA, no differences in constriction induced by NE were observed (Jovanovic et al., 1995). However, in another study, pregnancy decreased PE-mediated vasoconstrictor responses in guinea pig UtA (White et al., 1998). Chronic hypoxia does not alter blunted pregnancy-associated contractile response to adrenergic stimulation in UtA from guinea pigs (White et al., 1998) nor in human myometrial vessels (Lorca et al., 2019). Conversely, long-term hypoxia exposure decreases α-1 adrenergic receptor-mediated vasoconstrictor activity in pregnant sheep UtA by reducing adrenergic receptor densities (Hu et al., 1996) and diminishing the sensitivity of α-1 adrenergic receptor to inositol 1,4,5-trisphosphate (IP3)-mediated signaling (Hu et al., 1999). Complementary studies in the same animal model demonstrated that exposure to chronic hypoxia during pregnancy increases Ca^2+^ mobilization in response to α-1 adrenergic receptor agonist stimulus (i.e., NE), but reduces Ca^2+^ sensitivity in UtA myofilaments (Xiao and Zhang, 2004). This mechanism of diminished Ca^2+^ sensitivity in myofilaments may be associated, in part, with increased eNOS expression and subsequent cyclic GMP formation as shown in other vascular beds (McDaniel et al., 1992; Soloviev et al., 2004; Van Hove et al., 2009).

### 2.4 Protein kinase C (PKC)

Another mechanism that contributes to the regulation of vascular tone during pregnancy is signaling via PKC. Research in this pathway has been performed almost exclusively in ovine models, which are described here unless otherwise noted. In healthy, non-pregnant sheep, PKC activation induces sustained vasoconstriction in UtAs (Xiao and Zhang, 2002). During pregnancy, this vasoconstriction is attenuated by a reduction in PKC signaling and a consequent decrease in Ca^2+^ sensitivity in vascular SMCs (Xiao et al., 2006). Furthermore, PKC activation inhibits PE-dependent contractions by reducing the adrenergic-dependent [Ca^2+^]_i_ mobilization in normoxic pregnancies (Zhang et al., 2006). Actin polymerization, mediated by the PKC/ERK1/2 pathway, is responsible for the regulation of myogenic tone in UtA and is also decreased in UtAs during normal pregnancy, further decreasing vasoconstriction (Xiao et al., 2010b). In chronic hypoxia, the normal pregnancy-induced suppression of PKC signaling pathways is inhibited (Chang et al., 2009), increasing vascular tone and pro-constrictor response in the UtA via increases in basal Ca^2+^ sensitivity and actin polymerization (Xiao et al., 2010a; Xiao et al., 2012). This process has also been described in Sprague-Dawley rat resistance vessels exposed to prolonged stimulation with vasoconstrictors (Staiculescu et al., 2013). This maladaptation may be linked to the downregulation of ERα expression (Chang et al., 2010), since steroid hormones decrease PKC activity (Xiao et al., 2009).

### 2.5 K^+^ channels

Gestational hypoxia additionally induces PKC-mediated inhibition of UtA K^+^ channel activity, contributing to vascular dysfunction (Xiao et al., 2014). Large-conductance Ca^2+^-activated K^+^ (BK_Ca_) channels are responsible for the regulation of membrane potential in many cell types (Sancho and Kyle, 2021). BK_Ca_ channels are composed of pore-forming α subunits and their activity is regulated by several auxiliary subunits (β1-β4 and γ1-γ4) (Gonzalez-Perez and Lingle, 2019). In the vasculature, BK_Ca_ channels are mainly expressed in vascular SMCs and associated with β1 and γ1 subunits, which increase channel activity (Tanaka et al., 1997; Brenner et al., 2000; Evanson et al., 2014). In SMCs, BK_Ca_ channels hyperpolarize the plasma membrane in response to increases in [Ca^2+^]_i_, promoting vasodilation and opposing myogenic tone (Nelson et al., 1995). In the uterine circulation, pregnancy increases the activity of BK_Ca_ channels through regulation of its auxiliary subunits, leading to an increase in the diameter of the UtA, a decrease in myogenic tone, and increased UtA blood flow [reviewed by (Bresnitz and Lorca, 2022)]. Gestational hypoxia inhibits the increase in BK_Ca_ channel activity caused by estrogen in UtA during pregnancy (Chen et al., 2015). Several studies propose epigenetic modifications as a key mechanism in the hypoxia-dependent regulation of BK_Ca_ channel activity. Chronic hypoxia enhances the expression and activity of DNA methyltransferase (DNMT), resulting in excessive methylation of the promoter region of the BK_Ca_ channel β1 subunit (*KCNMB1*) and consequent suppression of its expression (Hu et al., 2017a). In one study, DNMT inhibitors effectively reversed the hypermethylation of the BK_Ca_ β1 promoter region caused by hypoxia, restored *KCNMB1* expression, and normalized channel activity, leading to enhanced UtA function (Hu et al., 2017a). Thus, hypoxia-induced epigenetic silencing is associated with reduced function of BK_Ca_ channels and impaired UtA adaptation to pregnancy.

MicroRNA-210 (miR-210) has emerged as a critical regulator in the hypoxia-induced repression of BK_Ca_ channels. miR-210 is a highly conserved small non-coding RNA that is involved in processes such as cell cycle and angiogenesis, and is upregulated in the UtA during gestational hypoxia (Huang et al., 2010; Ivan and Huang, 2014). miR-210 downregulates ten-eleven translocation methylcytosine dioxygenase 1 (TET1) expression (Hu et al., 2017b), a key enzyme involved in DNA demethylation (Guo et al., 2011b). This suppression of TET1 by miR-210 results in methylation of the *KCNMB1* promoter, impairing BK_Ca_ channel β1 subunit expression and function (Hu et al., 2017a; Hu et al., 2017b). Moreover, miR-210 also targets and downregulates the ryanodine type 2 receptor (RyR2) (Hu et al., 2021), a major regulator of Ca^2+^ release from the sarcoplasmic reticulum and modulator of vascular tone (Kassmann et al., 2019). Reduced RyR2 and BK_Ca_ channel β1 subunit expression lead to a decrease in Ca^2+^ sparks. In normal function, Ca^2+^ sparks that result from local Ca^2+^ release into the cytosol of the SMC activate the BK_Ca_ channel which, in turn, generate spontaneous transient outward currents (STOCs) in UtA (Zhuge et al., 2002; Song et al., 2021), contributing to membrane hyperpolarization and opposing vasoconstriction (Nelson et al., 1995). Thus, the hypoxia-elicited reduction in Ca^2+^ sparks increases uterine vascular myogenic tone. Studies in humans residing at high altitudes also have shown a SMC-specific reduction in BK_Ca_ channel activity, evidenced by a diminished sensitivity of myometrial arteries to the blocker tetraethylammonium (Fallahi et al., 2022). However, the mechanism(s) underlying the hypoxia-dependent regulation of BK_Ca_ in human uterine vasculature remain unknown.

Another Ca^2+^-activated K^+^ channel involved in regulating vascular tone and UtA adaptation during pregnancy is the small-conductance Ca^2+^-activated K^+^ channel (SK_Ca_). Specifically, SK_Ca_ types 2 and 3 are upregulated in the UtA during pregnancy (Zhu et al., 2013), facilitating vascular relaxation and adaptation. However, chronic hypoxia impedes this upregulation, leading to diminished SK_Ca_ channel activity and impaired myogenic reactivity in pregnant animals (Zhu et al., 2013), thus contributing to maladaptation of the uteroplacental circulation.

Hypoxia also leads to activation of K_ATP_ channels via a reduction in ATP levels. In human myometrial arteries from women with AGA pregnancies residing at high altitudes, there is an increased endothelium-dependent sensitivity of K_ATP_ channels to the blocker glibenclamide (Fallahi et al., 2022). This suggests that K_ATP_ channels are more active (or available) under chronic hypoxic conditions and could act as a compensatory mechanism in these uncomplicated human pregnancies at high altitudes. Interestingly, although these high-altitude pregnancies are AGA, they still show a non-pathological reduction in birth weight compared to lower altitudes (Lorca et al., 2019; Fallahi et al., 2022). However, in ovine models of high-altitude pregnancy, K_ATP_ channel activity is reduced in the UtA (Xiao et al., 2010c). These dissimilar observations between humans and sheep further highlight species-specific responses to hypoxia in the uterine vasculature. Moreover, the apparent redundancy of K^+^ channel activity promoting uterine vasodilation and increased blood flow may be an adaptive mechanism to preserve the uterine vascular adaptation to pregnancy under adverse conditions, such as chronic hypoxia.

### 2.6 Oxidative stress

Hypoxia elevates endoplasmic reticulum stress and oxidative stress in UtA, which also suppresses Ca^2+^ sparks/STOCs in the ovine pregnancy, increasing vascular tone (Hu et al., 2020). Additionally, mitochondrial dysfunction plays a crucial role in this process. Hypoxia and miR-210 enhance mitochondrial ROS (mtROS) production, inhibiting STOCs and contributing to increased myogenic tone (Hu et al., 2022). Notably, another target of miR-210, ten-eleven translocation methylcytosine dioxygenase 2 (TET2), which promotes DNA demethylation (Guo et al., 2011a), has been identified as a key regulator in this pathway. Downregulation of TET2 results in mitochondrial dysfunction and increased mtROS, thus decreasing STOCs and increasing myogenic contractions in the UtA, whereas overexpression of TET2 can mitigate these effects (Hu et al., 2023).

Furthermore, rats exposed to hypoxia during gestation and treated with a mitochondria-targeted antioxidant (MitoQ) showed an increase in maternal placental blood space and restoration of placental efficiency compared to untreated hypoxic animals (Nuzzo et al., 2018). Another study found that treatment with nanoparticle-encapsulated MitoQ reverses hypoxia-induced FGR and alleviates placental oxidative stress in a sex-dependent manner (Ganguly et al., 2021). Moreover, a recent study has shown a protective effect of MitoQ against UtA dysfunction and remodeling induced by chronic hypoxia during pregnancy in rats (Wang et al., 2024). Nevertheless, it should be considered that early gestation treatment with MitoQ could exacerbate the pre-eclamptic phenotype in mice by interfering with proper placentation (Yang et al., 2021).

Peroxisome proliferator-activated receptor gamma (PPARγ) is a hypoxia-sensitive ligand-inducible transcription factor with diverse functions, including the modulation of redox signaling in the vasculature (Kim and Yang, 2013). Inhibition of PPARγ during the second half of pregnancy decreased the vasodilator responses of the rat UtA, resulting in FGR (Gokina et al., 2013). In a mouse model of hypoxia-induced FGR, the pharmacological activation of PPARγ rescued fetal weight and prevented placental insufficiency (Lane et al., 2019). In the same rodent model, exposure to hypoxia during pregnancy increased endothelin-1-mediated UtA vasoconstriction, which was decreased by applying the selective PPAR-γ agonist troglitazone (TGZ) *ex vivo* (Lane et al., 2020a). In addition, UtA from hypoxic mice were more sensitive to TGZ-dependent vasodilation than UtA from normoxic animals (Lane et al., 2020a). These studies highlight PPARγ as a potential target to reverse the detrimental effects of oxidative stress and hypoxia during late gestation. Future studies should address the specific mechanism of PPARγ agonists in pregnant human uteroplacental circulation.

Resveratrol, an antioxidant drug, has also been widely studied in animal models of pregnancy. Resveratrol treatment improves fetal weight in a diabetic embryopathy model (Singh et al., 2011), induces UtA vasodilation in non-pregnant guinea pigs (Naderali et al., 2000), and reverses fetal death, but not the reduction in fetal weight, in a rat model exposed to hypoxia during pregnancy (Bourque et al., 2012). Moreover, resveratrol administered in the diet increases fetal weight and maternal UtA blood flow without changes in UtA vasoconstrictor or vasodilatory responses in a catechol-O-methyltransferase knockout model, which recapitulates characteristics of FGR and preeclampsia (Poudel et al., 2013). Sustained subcutaneous treatment with resveratrol in pregnant ewes increased UtA blood flow velocity and fetal growth (Darby et al., 2019). Subsequent studies demonstrated that acute administration of resveratrol in pregnant ewes has no significant effect on fetal hemodynamic improvements (Darby et al., 2023), indicating that successful treatment would require long-term intervention. Despite these benefits during pregnancy, resveratrol has not yet been sufficiently tested as a treatment for hypoxic human pregnancies and warrants further clinical study.

Melatonin, a neurohormone that upregulates antioxidant enzymes (Antolin et al., 1996), has been studied in several sheep models of FGR. Antenatal melatonin treatment has been shown to decrease brain injury in ovine ischemic-induced FGR offspring (Miller et al., 2014) and improve cerebrovascular function in ovine high-altitude-evoked FGR offspring (Candia et al., 2022). However, potential adverse effects have been reported in sheep exposed to chronic high-altitude hypoxia where antenatal treatment worsened FGR (Gonzalez-Candia et al., 2016). Moreover, antenatal melatonin treatment increased offspring mortality in rats (Singh et al., 2012) and failed to improve fetal weight or restore UtA vasodilatory function in an eNOS knockout mouse model of FGR (Renshall et al., 2018). A clinical trial in which melatonin was supplemented in women with early onset preeclampsia found no differences in the UtA pulsatility index but reduced the need for increasing antihypertensive drugs compared to the untreated women (Hobson et al., 2018).

N-acetylcysteine (NAC), an antioxidant likely acting as a precursor for glutathione synthesis (Samuni et al., 2013), partially reversed FGR in a guinea pig model and prevented fetal endothelial dysfunction (Herrera et al., 2017). Furthermore, NAC also restored vasodilatory responses in fetal arteries from chicken embryos exposed to hypoxia during development and in human chorionic arteries from FGR pregnancies (Krause et al., 2024). In addition, antenatal NAC treatment in FGR rats increased fetal brain weight at term without augmenting fetal weight (Chang et al., 2005).

Clinical studies have found limited or no effects of other antioxidants, such as vitamins C and E (Conde-Agudelo et al., 2011) and selenium (McDougall et al., 2023), in improving pregnancy outcomes (i.e., fetal weight). One clinical trial determined that using vitamins C and E in high concentrations exacerbates low birth weight (Poston et al., 2006).

Future efforts may focus on finding one or a combination of antioxidant treatment(s) for the improvement of maternal and fetal outcomes.

### 2.7 High-altitude ancestry

In human populations, high-altitude hypoxia has divergent effects on fetal growth and pregnancy outcomes depending on the ancestry of the individuals. Studies conducted in La Paz, Bolivia (elevation 3,600–4,100 m) revealed that women of Andean origin exhibit greater UtA diameter, cross-sectional area, and blood flow during pregnancy than those of European origin, resulting in improved uteroplacental oxygen delivery (Wilson et al., 2007). These physiological adaptations contribute to higher birth weights in newborns from Andean ancestry compared to their European counterparts at high altitudes (Julian et al., 2007). Interestingly, this is independent of maternal arterial oxygen content between the groups (Wilson et al., 2007; Julian et al., 2009). Women from lowland ancestry raised at high altitudes are not protected against the effects of high altitude on uteroplacental O_2_ delivery or reductions in birth weight, indicating this adaptation involves genetic, rather than developmental, factors (Julian et al., 2011). This may be due to a single nucleotide polymorphism (SNP) located near *PRKAA1*, the gene that encodes for the AMP-activated protein kinase (AMPK) α1 catalytic subunit, which is associated with higher UtA diameter and birthweight in altitude-adapted Andean populations (Bigham et al., 2014). Similar to Andeans, Tibetan populations living at high altitudes are protected from reduced birth weight compared to newcomers of Han ancestry (Moore et al., 2001). In addition, a high-altitude population in Ladakh, India (elevation 3,540 m), with mostly Tibetan ancestry, shows higher birth weights and larger UtA diameters than a low-altitude Indian population (Dolma et al., 2022). Furthermore, although no genome-wide significance of SNP was observed in Ladakhi populations, seven variants showed nominal associations in genes associated with birth weight (Bhandari et al., 2022). Overall, these associations underscore the critical role of UtA blood flow in fetal growth and highlight genetic factors that may enable high-altitude populations to better adapt to high-altitude hypoxia, reducing the incidence of adverse pregnancy outcomes.

### 2.8 AMP-activated protein kinase (AMPK)

Recent studies have highlighted the significant role of AMPK in modulating UtA blood flow and protecting against FGR under hypoxic conditions, including high altitude. AMPK acts as a metabolic sensor and is activated by ATP depletion, nutrient starvation, and hypoxia, among other stressors (Kim et al., 2016). AMPK induces vasodilatory responses by increasing NO bioavailability in ECs (Chen et al., 1999) and by decreasing [Ca^2+^]_i_ through sarcoplasmic/endoplasmic Ca^2+^-ATPase and BK_Ca_ channel activation in vascular SMC (Schneider et al., 2015). Following the identification of the *PRKAA1* SNP in a high-altitude human population (Bigham et al., 2014), a study in mice exposed to chronic hypoxia showed increased expression and activation of AMPK in UtAs (Skeffington et al., 2016). Accordingly, mice exposed to hypoxia during late pregnancy and treated *in vivo* with the AMPK agonist AICAR showed further increased UtA blood flow and partially attenuated reduction in fetal weight (Lane et al., 2020c). Furthermore, human myometrial arteries from women with AGA pregnancies residing at high altitudes showed increased AMPK-dependent vasodilation compared to low-altitude counterparts (Lorca et al., 2020). However, AMPK-dependent vasodilation was blunted in FGR pregnancies at high altitudes (Lorca et al., 2020). Thus, this vasodilatory response seems crucial for maintaining uteroplacental perfusion and supporting fetal growth in hypoxic environments. The limited research on AMPK activation in the human placenta at high altitude is mixed. In one study, high-altitude environments increased the activation of AMPK, measured as the ratio of total AMPK and its phosphorylated (Thr172) form (Lorca et al., 2021), whereas another study showed no change in AMPK activation (Yung et al., 2012). This discrepancy could be due to different sample sizes and/or altitude gradients between these studies. In mouse placenta, hypoxia during late pregnancy induces a reduction in phosphorylated AMPK (Lane et al., 2020b). Although the significance of the hypoxia-dependent regulation of placental AMPK for the regulation of uteroplacental blood flow remains unclear, taken together, these observations suggest an adaptive mechanism by which uterine vascular AMPK helps sustain the pregnancy-dependent rise in UtA blood flow under hypoxic conditions. There are potential therapeutic opportunities that may arise from these findings, and drugs that activate AMPK, such as metformin, are approved for certain pregnancy complications, such as gestational diabetes mellitus and polycystic ovary syndrome (Lautatzis et al., 2013). However, clinical randomized controlled trials have indicated that *in utero* exposure to metformin may lead to metabolic issues, increasing the risk of obesity in children (Hanem et al., 2018; Rowan et al., 2018). In addition, metformin can cross the placenta and affect fetal tissue (Charles et al., 2006), raising the likelihood of off-target effects. Thus, the wide-ranging effects of metformin discourage its use for specifically increasing uterine vasodilation in cases of reduced uteroplacental perfusion. Specific regulators of vascular AMPK targets could also be further studied to treat hypoxia-related vascular complications of pregnancy.

## 3 Hypoxia-induced regulation in fetoplacental circulation

The fetoplacental vascular bed is represented by umbilical cord arteries, umbilical vein, and the chorionic plate and villous blood vessels which include arteries, capillaries, and veins. Early studies aimed at addressing the acute effects of oxygen tension were performed in human placental cotyledons, in which acute hypoxia induces vasoconstriction, mainly in small caliber arteries (Howard et al., 1987; Hampl et al., 2002). This hypoxia-elicited vasoconstriction is partially mediated by a decrease in basal NO release by the endothelium (Byrne et al., 1997) and by an inhibition of voltage-gated K^+^ channels in the arteries of the chorionic plate (Hampl et al., 2002) with consequent activation of L-type Ca^2+^ channels (Jakoubek et al., 2006). Vasoconstrictor responses of the fetoplacental blood vessels to acute hypoxia appear to be very similar to that of the human pulmonary circulation (reviewed by (Ward and McMurtry, 2009)). Lowering O_2_ in pressurized chorionic plate veins induces vasoconstriction, whereas it evokes a moderate vasodilation in chorionic plate arteries (Wareing, 2012). Furthermore, nitrite-dependent vasodilation of chorionic plate arteries and veins is increased by acute hypoxia (Tropea et al., 2018). Chronic hypoxia due to residence at high altitudes also induces changes in the placental vasculature by increasing placental capillary density with decreased remodeling (Tissot van Patot et al., 2003). Future studies should investigate the effect of long-term hypoxia on the functional regulation of human fetoplacental circulation.

Studies using various animal models have been conducted to understand the impact of chronic hypoxia on the fetoplacental vascular bed. In rats, chronic hypoxia during pregnancy induces an increased vasoconstrictor response to angiotensin II and acute hypoxic challenges in fetoplacental vessels (Jakoubek et al., 2008). Furthermore, rat fetoplacental arteries exposed to chronic hypoxia during pregnancy showed increased collagen fiber accumulation, indicative of remodeling towards a pro-constrictor phenotype (Hvizdosova-Klescova et al., 2013). Similar to the observations in human placentas (Tissot van Patot et al., 2003), mouse and ewe models of gestational hypoxia showed an increase in the capillary network in the placenta (Parraguez et al., 2006; Cahill et al., 2018). This angiogenic response to hypoxia is also evident in the chorioallantoic membrane (Strick et al., 1991), the avian equivalent of the fetoplacental arterial circulation (Lindgren et al., 2010). Notably, the hypoxic chick embryo has been used as an animal model to study FGR independent of maternal hypoxic influences (Itani et al., 2018).

These studies in fetoplacental vessels showed that acute hypoxia-induced vasoconstriction of fetoplacental arteries reduces placental perfusion. However, capillary network expansion in the chronically hypoxic placenta acts as a local compensatory drive that potentially ensures the correct blood flow distribution and, therefore, oxygen supply to the fetus. Understanding this physiological adaptation may facilitate the identification of mechanisms that are compromised in pathological pregnancy conditions associated with impaired fetoplacental vasculature.

## 4 Conclusion

According to the developmental origins of adult diseases hypothesis (Barker’s hypothesis), pregnancy complications leading to decreased uteroplacental perfusion, placental dysfunction, and subsequent low birth weight affect early life and increase cardiometabolic risk in adulthood (Barker, 1990; de Boo and Harding, 2006). Since chronic hypoxia is a contributor to many vascular-associated pregnancy complications, such as FGR and preeclampsia, the mechanisms involved in the maternal and fetal vascular responses to hypoxic environments could highlight possible therapies to prevent or alleviate these complications. Particular attention should be given to the protective mechanisms observed in human populations residing at high altitudes (Julian et al., 2009; Bigham et al., 2014; Bhandari et al., 2022), as they could reveal novel targets for improving uteroplacental and fetoplacental perfusion. The development of preclinical models of chronic hypoxia have also been important for the testing of new drugs and therapies. For example, antioxidants and other metabolism-modifying drugs have been described in animal models to attenuate the effects of hypoxia on maternal vasculature, alleviating the development of pregnancy complications (Lane et al., 2019; Lane et al., 2020c; Wang et al., 2024). Future studies aiming to develop treatments targeted at uteroplacental and/or fetoplacental vasculature may prevent the non-specific adverse vascular effects observed in previous clinical trials using broad vasodilators (Pels et al., 2020). Additionally, the impact of hypoxia on placental physiology and its link to pregnancy complications has been extensively studied, reviewed by (Colson et al., 2021), and this information should also be taken into account when developing therapies. Thus, using animal models of impaired placental function can enhance our understanding of pregnancy complications associated with vascular issues.
